# The complete mitochondrial genome of *Pareuchiloglanis myzostoma* (Teleostei, Siluriformes)

**DOI:** 10.1080/23802359.2019.1676173

**Published:** 2019-10-18

**Authors:** Lili Cui, Haitao Gao, Xiangjun Miao, Mingli Li, Guanghua Li, Gefeng Xu, Junjie Wu, Wei Hu, Shaoxiong Lu

**Affiliations:** a College of Animal Science and Technology, Yunnan Agricultural University, Kunming, PR China;; b Yunnan Academy of Fishery Sciences, Kunming, PR China;; c Heilongjiang River Fisheries Research Institute, Chinese Academy of Fishery Sciences, Harbin, PR China

**Keywords:** *Pareuchiloglanis myzostoma*, mitochondrial genome, phylogeny

## Abstract

*Pareuchiloglanis myzostoma* is a key-listed protected indigenous fish species in Nujiang, Yunnan, China. In this study, we firstly reported the complete mitochondrial genome of *P. myzostoma*, which was 16,584 bp in length, containing 13 protein-coding genes, 22 transfer RNAs (tRNAs), 2 ribosomal RNA genes (rRNAs), and a non-coding control region (D-loop). The overall base composition of *P. myzostoma* was 30.7% for A, 24.2% for T, 16.0% for G, and 29.1% for C. Phylogenetic analysis showed that all Sisoridae species clustered together formed a monophyletic group. This work would provide a set of useful data on further molecular evolution studies of this precious species.

*Pareuchiloglanis myzostoma* (Norman 1923), commonly known as flat-headed fish, which belong to order Siluriformes, family Sisoridae, and genus Pareuchiloglanis, is a unique and rare fish in Nujiang, Yunnan Province (Zhou et al. 2005). It has been listed in the Chinese Vertebrate Red List (Jiang et al. 2016) and the Nujiang Key Conservation List of Indigenous Fish (http://yunnan.mofcom.gov.cn).

In this study, we determined the complete mtDNA sequence of *P. myzostoma*. Samples were collected from Nujiang county of Yunnan Province in China (26°53′N; 99°07′E). The specimen is stored in the Specimen Museum of Yunnan Academy of Fishery Sciences and its accession number is 20180903001. The sequencing results were assembled using NOVOPlasty (https://github.com/ndierckx/NOVOPlasty). Genomes were predicted using the MitoAnnotator (http://mitofish.aori.u-tokyo.ac.jp/annotation/input.html). The transfer RNA (tRNA) genes were identified using the programme tRNAscan-SE (Lowe and Eddy 1997). The locations of protein-coding genes were determined by comparing with the corresponding known sequences of other Pareuchiloglanis fish species.

The whole mitochondrial genome length of *P. myzostoma* was 16,584 bp in length (GenBank accession number MK617319). It consisted of a non-coding control region (D-loop), 13 protein-coding genes, 2 ribosomal RNA genes (rRNAs), and 22 tRNAs. The contents of A, C, G, and T were 30.7%, 29.1%, 16.0%, and 24.2%. The percentage of G + C content was 45.1%, which was lower than that of A + T content (54.9%). To demonstrate the phylogenetic position of *P. myzostoma*, we performed MEGA version 7.0 (Arizona State University, Phoenix, State of Arizona, United States of America) (Kumar et al. 2016) to align all selected sequences and construct a neighbour-joining tree containing complete mitochondrial genome DNA of 23 species. The results from the phylogenetic analysis revealed that all Sisoridae species clustered together formed a monophyletic group and *P. myzostoma* has a close relationship with *Pseudexostoma yunnanensis*. However, the genus *Pareuchiloglanis* was not a monophyletic group because it was also clustered with other genus *Bagarius, Pseudexostoma*, *Creteuchiloglanis*, and *Euchiloglanis* (Figure 1).

**Figure 1.**
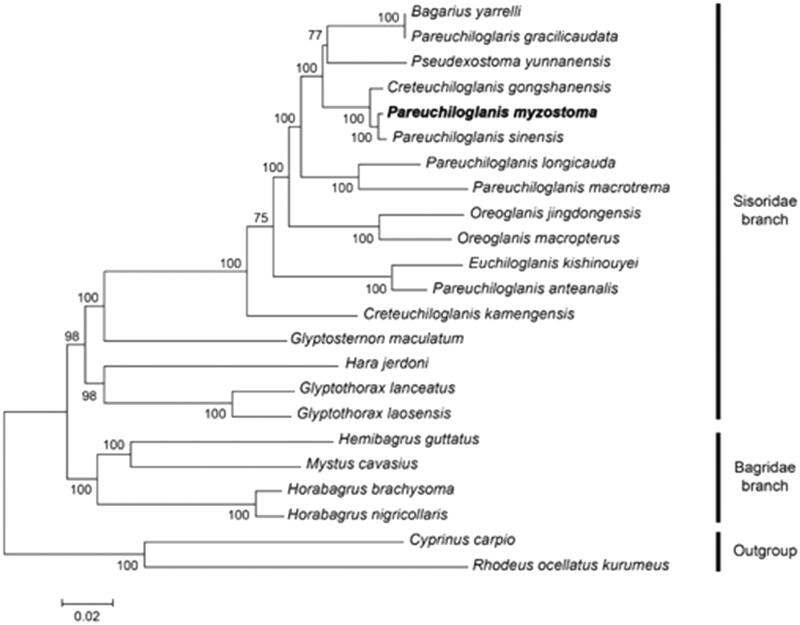
A neighbour-joining (NJ) tree of the 23 species from *Siluriformes* was constructed based on complete mitochondrial genome data. The analysed species and corresponding NCBI accession numbers are as follows: *Bagarius yarrelli* (KP342264.1), *Pareuchiloglanis gracilicaudata* (JQ026237.1), *Pseudexostoma yunnanensis* (JQ026258.1), *Creteuchiloglanis gongshanensis* (KP872697.1), *P. myzostoma* (MK617319), *Pareuchiloglanis sinensis* (KJ637323.1), *Pareuchiloglanis longicauda* (KP872693.1), *Pareuchiloglanis macrotrema* (KP872694.1), *Oreoglanis jingdongensis* (KP872691.1), *Oreoglanis macropterus* (JQ026261.1), *Euchiloglanis kishinouyei* (JQ026252.1), *Pareuchiloglanis anteanalis* (KP872692.1), *Creteuchiloglanis kamengensis* (JQ026253.1), *Glyptosternon maculatum* (JQ026251.1), *Hara jerdoni* (AP012012.1), *Glyptothorax lanceatus* (NC_039895.1), *Glyptothorax laosensis* (NC_039702.1), *Hemibagrus guttatus* (KJ584373.1), *Mystus cavasius* (KU870465.1), *Horabagrus brachysoma* (KU870467.1), *Horabagrus nigricollaris* (MG986722.1), *Cyprinus carpio* (NC_001606.1), and *Rhodeus ocellatus kurumeus* (AB070205.1).
